# Synergistic Combination of Gemcitabine and Dietary Molecule Induces Apoptosis in Pancreatic Cancer Cells and Down Regulates PKM2 Expression

**DOI:** 10.1371/journal.pone.0107154

**Published:** 2014-09-08

**Authors:** Archana Pandita, Bhupender Kumar, Siddharth Manvati, Samantha Vaishnavi, Shashank K. Singh, Rameshwar N. K. Bamezai

**Affiliations:** 1 School of Biotechnology, Shri Mata Vaishno Devi University, Katra, J&K, India; 2 School of Life Sciences, Jawaharlal Nehru University, New Delhi, India; 3 Indian Institute of Integrative Medicine, CSIR, Jammu, J&K, India; University of South Alabama Mitchell Cancer Institute, United States of America

## Abstract

Gemcitabine, an effective agent in treatment of cancer of pancreas, has undergone failures in many instances after multiple cycles of therapy due to emergence of drug resistance. Combination of dietary compounds with clinically validated drugs has emerged as an effective therapeutic approach to treat pancreatic tumors, refractory to gemcitabine therapy. In order to optimize a possible synergistic combination of Gemcitabine (GCB) with dietary molecules, Betuilnic acid (BA) and Thymoquinone (TQ), stand-alone IC_50_ dose of GCB, BA and TQ was calculated for pancreatic cancer cell lines. Fixed IC_50_ dose ratio of the dietary molecules in combination with reduced IC_50_ dose of GCB was tested on GCB resistant PANC-1 and sensitive MIA PaCa-2 cells for synergism, additive response and antagonism, using calcusyn. Combination index (CI) revealed that pre-treatment of BA and TQ along with GCB synergistically inhibited the cancer cell proliferation in *in-vitro* experiments. Pyruvate kinase (PK) M2 isoform, a promising target involved in cancer cell metabolism, showed down-regulation in presence of TQ or BA in combination with GCB. GCB with BA acted preferentially on tumor mitochondria and triggered mitochondrial permeability transition. Pre-exposure of the cell lines, MIA PaCa-2 and PANC-1, to TQ in combination with GCB induced apoptosis. Thus, the effectiveness of BA or TQ in combination with GCB to inhibit cell proliferation, induce apoptosis and down-regulate the expression of PKM2, reflects promise in pancreatic cancer treatment.

## Introduction

Attempts have been madeto improve upon the efficacy of clinically validated drugs in combination regimen to enhance their response towards refractory tumors, including pancreatic cancer [1]. Beside 5-Flurouracil, Gemcitabine (GCB), a widely used chemotherapeutic drug for pancreatic cancer, has shown failures after multiple cycles of therapy because of the emergence of drug resistance [2], [3]. Treating pancreatic tumors that are refractory to gemcitabine therapy is a challenge for oncologists. Efforts,as in other cancers, to target the key processes, such as carcinogen metabolism, cell division, differentiation, apoptosis, in pancreatic cancer development, has generated interest in dietary phytochemicals for potential cancer chemoprevention. A mechanistic link between diet and pancreatic cancer comes from its long-recognized interrelationship [4]. One such dietary agent, which could be used in combination with GCB for treatment of pancreatic cancer, is Betulinic acid (BA), a naturally occurring penta-cyclic-triterpene with a variety of biological activities including potent antitumor properties [5]. BA is contained in the outer bark of various plants throughout the plant kingdom, including white-barked birch trees [6], with anti-inflammatory, anti-viral, and anti-neoplastic activities [7]. Anticancer activity of BA has been linked to its ability to directly trigger mitochondrial membrane permeabilization, a central event in the apoptotic process, raising the hope to bypass the resistance to conventional chemotherapeutics [6]. Thymoquinone (TQ), another potential anticancer-nutraceutical agent, is a bioactive compound derived from black seed (*Nigella sativa*) oil. In folklore medicine, consumption of TQ seed has been associated with diverse therapeutic benefits in bronchial asthma, dysentery, headache, gastrointestinal problems, eczema, hypertension and obesity [8]. In the context of cancer, TQ is reported to exhibit anti-proliferative effects on cell lines, derived from breast, colon, ovary, larynx, lung, myeloblastic leukemia and osteosarcoma [9]–[15]; and anti-metastasis effect in humanpancreatic cancer. It has been shown to suppress the migration and invasion of PANC-1 cells in a dose-dependent manner [16] and down-regulate NF-kappa B and MMP-9 expression. Previous studies have also shown the biological activity of thymoquinone (TQ) in pancreatic cancer cells*in vitro*. This has revealed its chemo-sensitizing effect after the pre-exposure of cells to TQ (25 µmol/L) for 48 hrs, followed by gemcitabine or oxaliplatin, resulting in 60% to 80% growth inhibition as compared to 15% to 25% of inhibition with gemcitabine or oxaliplatinalone [17].

Cancer cells, predominantly dependent on the reprogramming of their metabolic needs, consume more glucose and produce a large amount of lactate even in a well–oxygenized environment; the process termed as aerobic glycolysis or “Warburg effect” [18], [19]. While normal differentiated cells maximize ATP production by mitochondrial oxidative phosphorylation of glucose under normoxic conditions, cancer cells generate much less ATP from glucose by aerobic glycolysis. Despite being less efficient in ATP production, glycolysis is a much more rapid process in cancer cells [20], [21]. Here pyruvate kinase (PK) catalyzes the last reaction with transfer of a high-energy phosphate group from phospho-enolpyruvate (PEP) to ADP, producing ATP and pyruvate which is reduced to lactate by lactate dehydrogenase (LDH) in the cytosol. Pyruvate kinase (PK) consists of four isoforms, of which PKM2 expresses predominantly in cancer cells [22]. These are: colon [23], renal cell [24], lung [25] and others. PKM2 has been shown to act as a marker for: renal cell carcinoma (RCC) [26], [27], testicular cancer [28], breast cancer [29], urological tumors [30], lung carcinoma, cervical cancer, and gastrointestinal tumors [31]; and with a possible detection in the feces of patients with gastric and colorectal cancers [32]. In recent past, mass spectrometry has further demonstrated a predominant presence of PKM2 in: RCC, bladder carcinoma, hepatocellular carcinoma, colorectal cancer, lung carcinoma, and follicular thyroid adenoma [33].

In this study, we investigated the effect of BA or TQ with GCB, independently and in combination, on human adenocarcinoma cells, MIA PaCa-2 and PANC-1, to induce cell death. Underlying mechanism of their action, especially with respect to PKM2 expression and activity and mitochondrial permeability transition was assessed.

## Materials and Methods

### Cell Lines, maintenance and reagents

Human pancreatic cancer cell lines, PANC-1 and MIA PaCa-2 were purchased from American Type Culture Collection (Manassas, VA). Cells were cultured in DMEM supplemented with 10% fetal bovine serum at 37°C in a humidified 5% CO_2_ atmosphere. Glutamine, HEPES or sodium pyruvate supplements, were added to maintain proper cell growth. Betulinic acid (BA), Thymoquinone (TQ), Gemcitabine (GCB), dimethylthiazole-2-yl-2, 5-diphenyl-tetrazolium-bromide (MTT), Rodhamine-123 (Rh-123), Propidium Iodide (PI) and other chemicals (Sigma Chemical Co, USA); along with Annexin-V-FITC apoptosis detection kit (Cayman Chemical Company), antibodies against: Pro-Caspase-3, Beta-actin, PKM2, poly-ADP-ribose-polymerase (PARP) (Cell Signaling), were procured and used in the study.

### Treatment profile and Cell viability assay

PANC-1 and MIA PaCa-2 cells were grown in T-75 flasks. After 80% confluency, cells were trypsinized and centrifuged at 100×g for 5 min. The cell pellet was suspended in DMEM medium; and 2×10^5^ cells/well dispensed in Nunclon-96-well flat bottom plates to attach for 24 hrs. Cells were exposed to different concentrations of BA, TQ and GCB, alone and in combinations. In combination experiments, the cells were first treated and sensitized with BA or TQ for 24 hrs, followed by exposure to GCB for next 24 hrs and incubated in CO_2_ incubator at 37°C. For cell viability assay, MTT solution (20 µl of 2.5 mg/ml in DMEM) was added to each well and the culture plates stirred gently, followed by incubation in CO_2_ incubator at 37°C for 3 hrs. The supernatant was aspirated and MTT–formazon crystals dissolved in 150 µl DMSO. Plates were again incubated at 37°C and stirred for 10 min on a plate shaker. The absorbance of DMSO dissolved MTT–formazon crystals was measured at 570 nm. Chemo-sensitivity values were expressed as % cell viability of the drug concentration that inhibited 50% cell growth; and the IC_50_ values calculated from the concentration-effect relationships, using Graph Pad prism Version 5.00.

### Analysis of combined drug effects

Drug synergy was determined by the isobologram and combination-index methods, derived from the median effect principle of Chou and Talalay [34], using the CalcuSyn software (Biosoft, Version 2.1). Data obtained from the growth inhibitory experiments was used to perform these analyses. The isobologram method is a graphical representation of the pharmacologic interaction; and is formed by selecting a desired fractional affected cell kill (Fa). A straight line was drawn to connect the Fa points plotted against experimentally used fixed ratio combinations of drug (GCB) and the dietary molecule (BA or TQ) on X- and Y-axes to generate isobolograms. Combination data points that fell on the line represented an additive drug-drug interaction; whereas, data points that were below or above the line represented synergism or antagonism, respectively. The combination-index (CI), a numerical value, was calculated by the formula [35]: 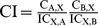 Where, C_A, x_ and C_B, x_, were the concentrations of drug A anddrug B, used in combination to achieve x% drug effect. IC_x, A_ and IC_x, B_ were the concentrations of individual agents to achieve the same effect.

The combination-index (CI) method is a mathematical and quantitative representation of a two-drug pharmacologic interaction. Using data from the growth inhibitory experiments and computerized software, CI values were generated over a range of Fa levels from 0.05–0.90 (5%–90% growth inhibition). A CI of 1 indicated an additive effect between the two agents, whereas a CI < 1 or CI > 1 indicated, synergism or antagonism, respectively.

### Flow cytometric analysis for detection of apoptosis

1×10^5^ cells/ml/well of MIA PaCa-2 and PANC-1 were treated with BA, TQ, and GCB alone and in combination for 48 hrs. Cells were washed and stained with Annexin-V-FITC antibody and PI, as per the instructions of the manufacturer. Flow-cytometric dot plot assay was performed and cells scanned for fluorescence intensity in FL-1 (FITC) and FL-2 (PI) channels. The fraction of cell population in different quadrants was analyzed, using quadrant statistics. Cells in the lower right quadrant, represented apoptosis; and in the upper right quadrant, necrosis or post apoptotic necrosis [36].

### Measurement of mitochondrial membrane potential

1×10^5^ cells/ml/well of MIA PaCa-2 and PANC-1 cells were treated with BA, TQ, and GCB alone and in combination in 12-well plate for 48 hrs. In the last 1 hr of incubation, before termination of the exposure, cells were stained with rhodamine-123 (5 µg/ml). Cells were washed in PBS and centrifuged at 100×g for 5 min and suspended in PBS. To detect the changes in mitochondrial trans-membrane potential (ψ), as a result of mitochondrial perturbation, a decrease in fluorescence intensity was analyzed flow-cytometrically on FL-1channel [37].

### Preparation of whole cell lysate and immunoblotting

Cells, (2×10^6^/6 mL medium/60 mm tissue culture plate) exposed to BA, TQ, and GCB alone and in combination after 48 hrs of treatment, were harvested by trypsinization and washed with PBS.The pellet obtained was lysed with ice cold lysis buffer (50 mMTris pH 8.0, 150 mMNaCl, 5 mM EDTA, 1% v/v Nonidet P-40, 1 mM PMSF and 1% (v/v) protease inhibitor cocktail) and kept for 30 min on ice with intermediate tapping after every 5 minutes. The lysates were centrifuged at 12000 g for 10 min at 4°C and supernatant collected for immunoblotting [38]. The supernatant so collected was quantified using bicinchoninic acid assay (BCA assay) kit from Thermo Scientific. For Western blot analysis, 50 µg total protein was resolved on SDS–PAGE at 60 V and then electro transferred to PVDF membrane overnight at 4°C under a 30 V current. Non-specific protein binding was blocked with 5% non-fat milk in Tris-buffered saline, containing 0.1% Tween-20 (TBST), for 1 hr at room temperature. The blots were probed with respective primary mouse/rabbit/goat anti human antibodies for 2 hrs and washed three times with TBST. The blots were then incubated with horse-radish peroxidase conjugated secondary antibodies for 1 hr, washed again three times with TBST and signals detected, using ECL plus chemi-luminescence kit on an X-ray film [38].

### PKM2 activity assay

PKM2 activity was measured spectro-photometrically (UV-1800, Shimadzu, Japan), using NADH/lactate dehydrogenase (LDH) coupled assay as described earlier [39].

### Statistical analysis

Data of the experiments carried out in triplicate was expressed as mean+/-SD, unless indicated otherwise. Comparisons were made between control and treated groups, using One-way ANOVA (Dunnett or Tukey) and P values <0.01 were considered significant.

## Results

### *In vitro*combination studies of gemcitabine with betulinic acid and thymoquinone

**Figure 1:**
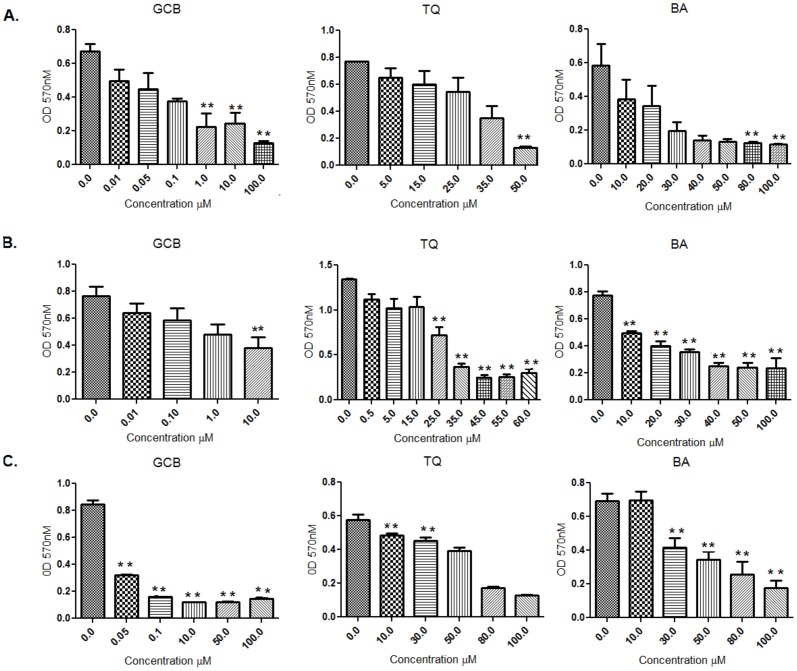
IC_50_ of Gemcitabine, Thymoquinone and Betulinic acid at 48 hrs. *IC_50_* *value was calculated on* *Human pancreas adenocarcinoma* cell lines (A) MIA PaCa-2 (B) PANC-1 and (C) Normal cell line FR2: For individual treatment of Gemcitabine, Thymoquinone and Betulinic acid for 48 hrs (**, significant as compaired to control at p value<0.01, n = 3) in-between the three cell lines.

**Figure 2:**
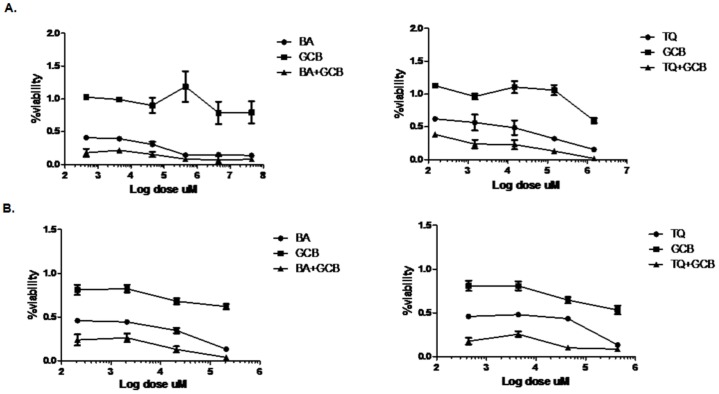
Effect of Betulinic acid, Thymoquinone and Gemcitabine alone and (in combination) on growth of human pancreatic tumor cell lines. *(A) MIA PaCa-2 and (B) PANC-1 cells were exposed to graded concentrations of Betulinic acid, Thymoquinone or Gemcitabine either alone or in combination at a fixed dose ratio of Betulinic acid vs. Gemcitabine and Thymoquinone vs. Gemcitabine for 48 hrs.(Gemcitabine* *having exposure for 24 hrs only in case of combination), Gemcitabine (squares),* *Betulinic acid and Thymoquinone* (circle) Gemcitabine+ betulinic acid and Gemcitabine +Thymoquinone (triangle).(Mono therapy (BA, GCB, TQ) Versus Combinations(CI_(GCB:BA)_, CI_(GCB:TQ)_)(**, significant difference at p value <0.01, n = 3).

**Figure 3:**
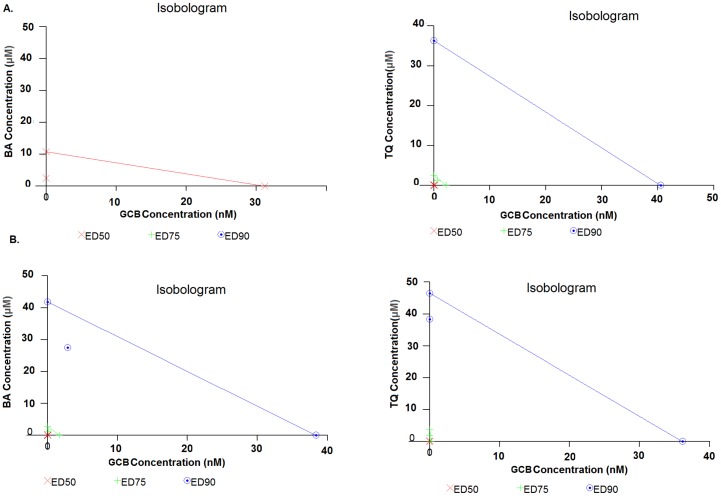
Isolobolograms of the combinations of Gemcitabine with Betulinic acid or Thymoquinone. *(A and B) Showing isolobolograms analysis of the combination of gemcitabine, Betulinic acid or Thymoquinone in, (A) MIA PaCa-2 cells with a fixed drug ratio 1∶0.01 of GCB:BA and GCB:TQ* (B) PANC-1 cells with a fixed drug ratio 1∶0.2 of GCB:BA and GCB:TQ. The individual doses of gemcitabine, Betulinic acid and Thymoquinone, to achieve 90% (straight line) growth inhibition (Fa  =  0.90), 75% (hyphenated line) growth inhibition (Fa  =  0.75), and 50% (crosses) growth inhibition (Fa  =  0.50) were plotted on the x- and y-axes. Combination index (CI) values calculated using Calcusyn software is represented by points above (indicating- antagonism between drugs) or below the lines (indicating- synergy). (X symbol) ED50, (plus sign) ED75 and (open dotted circle) ED90 (Monotherapy (BA, GCB, TQ) versus combinations (CI_ (GCB: BA)_, CI_ (GCB: TQ)_) (***, significant difference at p value<0.001, n = 3).

The chemo-sensitivity in pancreatic cancer cell lines, MIA PaCa-2 and PANC-1, towards the drug GCB and the dietary molecules, BA or TQ, was reflected in cell growth inhibition, calculated by IC_50_ (50% growth inhibition) at 48 hrs; and compared initially with the values obtained in a control cell line, FR2. The IC_50_ values obtained from independent treatment in the three cell lines, MIA PaCa-2, PANC-1 and FR2, are provided in detail in Table 1(BA: 25±0.9 µM, 23.5±3.5 µM, 50±1 µM; TQ 36± 0.28 µM, 23±2 µM and 77±2 µM; GCB 1±0.66 µM, 5.5±2.5 µM and <0.025 µM). Results were analysed with One-way Annova–Dunnett showing a significant (p value<0.01) effect among treated, in a dose dependent manner (**Figure 1**). Based on these results, a fixed drug ratio was selected (GCB:BA or GCB:TQ) over a range of drug concentrations and used in further experimentations (**Figure 2**). Combination-index (CI) was used to analyse and confirm synergism observed after pre-treatment of cells with dietary molecule, followed by the exposure to GCB for 48 hrs (GCB:BA and GCB:TQ). The CI values, providing a quantitative measure of the degree of drug interaction, were calculated using CalcuSyn software, at Fa values of 0.50, 0.75, and 0.90 (Table 2). Isobolograms constructed for these values, representing 50%, 75% and 90% growth inhibition respectively, both for MIA PaCa-2 and PANC-1 cells (**Figure 3**) and Fa-CI plot, is provided as **Figure S1**. Table 3 depicts the CI values at Fa of 0.50 to 0.90 for the dietary molecules (BA or TQ) and drug (GCB). A comparison of monotherapy (BA, GCB, TQ) with combination (CI_(GCB:BA)_ and CI_(GCB:TQ)_) showed a significant difference at p value<0.01. ED values of the isolobologram for MIA PaCa-2 and PANC-1 are mentioned in Table S1. In MIA PaCa-2 cells, sensitized with BA and followed with GCB exposure, the CI _(GCB: BA)_ values obtained at doses (6.25∶0.082 and 25∶0.33) were 0.519, 0.889. With TQ, the CI _(GCB: TQ)_ at doses (4.5∶0.082; 9∶0.15 and 36∶0.66) showed the values of 0.721, 1.050, 0.681. However, in PANC-1 the CI _(GCB:BA)_ at (5∶1.3 and 20∶5.2) doses showed 0.766, 0.429 and with TQ CI_(GCB:TQ)_ at doses (6.25∶1.3 and 25∶5.3), the values were 0.736, 0.371. The CI values of <1;< 0.5 and >1, represented synergism, strong synergism and antagonistic effect, respectively. The synergism was observed across a broad range of concentrations in both the studied pancreatic tumor cell lines. Synergistic drug combination i.e. CI (the combination index) of CI_(GCB:BA)_ (25+0.33) and CI_(GCB:TQ)_ (36+0.66) in MIA PaCa-2cell line and CI_(GCB:BA)_ (20+5.25) and CI_(GCB:TQ)_ (25+5.25) in PANC-1, representing the Fa value 0.75 (showing 75% inhibition), were used in rest of the assays. However, the normal cell line used in the study (FR2), for comparison as a control, showed inhibition at the Fa value of 0.75 to 0.90. Our results showed that the impact of the dietary molecules (BA or TQ) in the normal cells was observed at a high concentration; while only a minimal dose of GCB was required for the inhibition of cell proliferation. In case of combination studies, the total exposure of gemcitabine to the cancer cells was only for 24 hrs.

**Table 1:** Mean IC_50_ Value of drug (GCB) and dietary molecules (BA and TQ) on three cell lines (MIA PaCa-2; PANC-1; FR2) at 48 hrs.

Cell line	Dietary molecule/Drug	IC_50_ (µM) ± SEM
**MIA PaCa-2**	**BA**	25±0.9
	**TQ**	36± 0.28
	**GCB**	1±0.66
**PANC-1**	**BA**	23.5±3.5
	**TQ**	23±2
	**GCB**	5.5±2.5
**FR2**	**BA**	50±1
	**TQ**	77±2
	**GCB**	< 0.025

IC_50_ concentration of the drug (GCB) and Dietary molecules (BA and TQ) in **µ**M concentration causing 50% growth inhibition of two human pancreatic cancer cell lines(MIA PaCa-2 and PANC-1) and non cancer cell line(FR2) after 48 hrs of treatment.

**Table 2:** Combination Index (CI) at ED_50_, ED_75_, ED_90_ values of Dietary with drug combination on two pancreas cancer cell lines.

Cell line	Dietary: Drug	ED_50_	ED_75_	ED_90_
**MIA PaCa-2**	GCB: BA	0.24061	13.82921	798.34755
	GCB: TQ	0.19091	0.85714	3.91836
**PANC-1**	GCB: BA	0.47653	0.58427	0.73317
	GCB: TQ	0.42302	0.54244	0.82430

The CI Values at a Fa value of 0.5, 0.75, 0.90 for islobologram (Figure 3) were calculated with the CalcusynVersion 2.1 software**.**

**Table 3:** CI values of Dietary molecules (BA and TQ) in combination with Drug (GCB).

CI For experimental values (MIA PaCa-2 cell line)			
**BA**	**GCB**	CI*	**Mode**
6.25	0.0825	0.519	++
25	0.33	0.889	+
**TQ**	**GCB**	CI *	**Mode**
4.5	0.0819	0.721	+
9	0.153	1.05	-
36	0.6552	0.681	+

CI For experimental values (PANC-1 Cell line)			
**BA**	**GCB**	CI*	**Mode**
5	1.3	0.766	+
20	5.2	0.429	++
**TQ**	**GCB**	CI*	**Mode**
6.25	1.3	0.736	+
25	5.3	0.371	++

CI*(combination index) obtained from the Fa value which denotes the fraction affected (e.g., Fa of 0.5 is equivalent to a 50% reduction in cell growth). The CI value less than 1 shows synergism, equal to 1 show a additivism while greater than 1 shows antagonism.(++)strong synergism,(+)synergism,(-) antagonism. Cells were exposed to a fixed ratio dose concentration of BA or TQ either alone or along with combination at a dose ratio of GCB verses BA and GCB verses TQ for 48 hrs as shown in Figure 2.

### Loss of mitochondrial membrane potential

**Figure 4:**
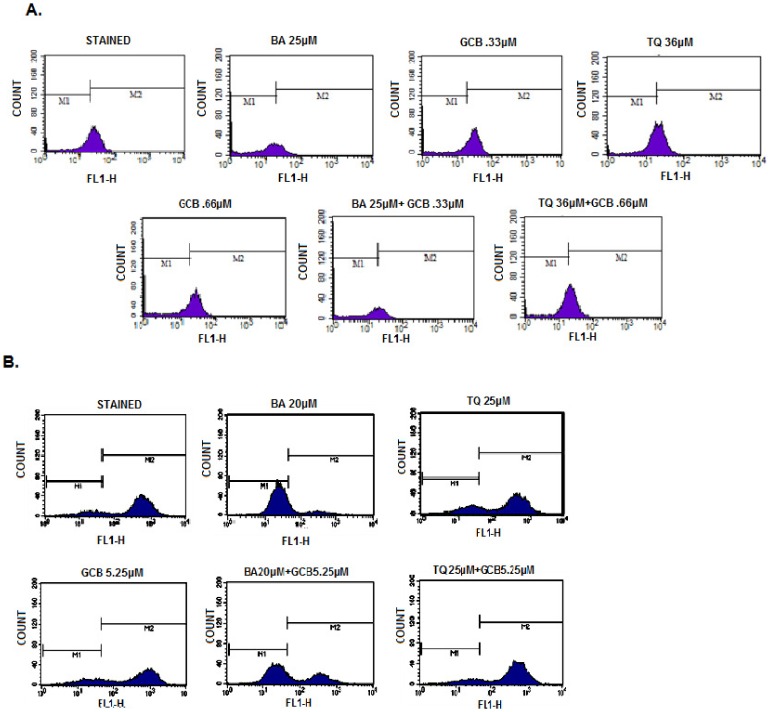
Effect on Mitochondrial membrane potential by Gemcitabine, Betulinic acid and Thymoquinone alone and in combinations. *(A) MIA PaCa-2: Betulinic acid* alone and in combination CI _(GCB:BA)_ with Gemcitabine induced loss of mitochondrial membrane potential (ψ) that is not observed in Thymoquinone and Gemcitabine neither alone nor in combination CI _(GCB:TQ)_. Cells were stained with Rhodamine-123 (5 mg/mL) for 1 hr and analyzed in FL-1 channel of flow cytometer. Data in all figures are representative of one of three similar experiments. (B) Similar studies were carried out in PANC-1 cell line, Betulinic acid alone induces more loss of mitochondrial membrane than in combination with Gemcitabine CI_(GCB:BA)_ also no loss in mitochondrial membrane was observed when treated with Thymoquinone and gemcitabine alone or in combination CI_(GCB:TQ)_ for 48 hrs.(Control verses Monotherapy (BA, TQ, GCB) and Control verses Combinations (CI_(GCB:BA)_, CI_(GCB:TQ)_),(***, significant difference at p value<0.001, n = 3).

BA or TQ pre-treated MIA PaCa-2 and PANC-1 cells along with GCB, in combination and independently, when analyzed for Rh-123 uptake by flow cytometery, displayed a loss of 11% and 17% Mitochondrial Membrane Potential (MMP), in MIA PaCa-2 cells treated with 0.33 µM and 0.66 µM of GCB, respectively. While in combination, CI_ (BA:GCB)_, thedecrease was 77%when compared to the control (p value<0.001) (**Figure 4A**). However, PANC-1 cells, which are relatively resistant to GCB when compared to MIA PaCa-2, showed a decrease of 74% with BA alone and in combination, CI_(GCB:BA)_, the decrease was up to46%, as compared to the control (p value <0.001). The treatment with the individual dose of GCB i.e. 5.25 µM, showed a decrease up to 13% (**Figure 4B**). TQ alone or in combination with GCB did not show any change in MMP in both the cell lines. Apparently, BA equi-potently targeted mitochondrial functions than TQ and GCB, in both cell types. In the untreated control cells, almost all cells were bio-energetically active, as evidenced by high Rh-123 fluorescence.

### Flow cytometric analysis- scoring of apoptotic cells, treated with BA, TQ and GCB, alone and in combination

**Figure 5:**
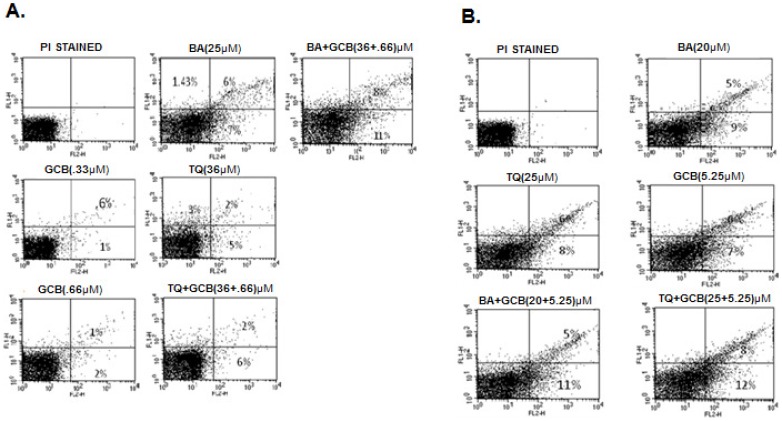
Flow cytometric analysis of apoptotic cell population. *(A) MIA PaCa-2 cells (1×10^5^) were incubated with indicated concentrations of Betulinic acid, Thymoquinone and Gemcitabine* *alone and in combination CI_(GCB:BA)_, and CI_(GCB:TQ)_ for* 48 hrs and analyzed for annexinV-FITC/PI staining to determine apoptotic cell populations as described in Materials and Methods Section. (B) Similar studies were carried out with in PANC-1 cells after treatment with indicated doses of Betulinic acid, Thymoquinone and Gemcitabine alone and in combination CI_(GCB:BA)_, and CI_(GCB:TQ)_ for 48 hrs.(Control versus monotherapy (BA, TQ, GCB) and control versus combinations (CI_(GCB:BA)_, and CI_(GCB:TQ)_), (***, significant difference at p value<0.001, n = 3).

The enhanced cytotoxicity by BA or TQ pre-treatment induced apoptosis was a question to be answered. BA or TQ pre-treated cells along with GCB, in combination and individually, produced dose dependent increase in apoptotic and post apoptotic cell population in both the pancreatic cancer cell lines, MIA PaCa-2 and PANC-1. In MIA PaCa-2, independent treatment with BA (25 µM), TQ (36 µM) and GCB (0.33 µM and 0.66 µM) resulted in 7%, 5%, 1% and 2% rate of apoptosis, respectively. In combination, CI _(GCB:BA)_, produced 11% and CI _(GCB:TQ)_ produced 6% (p value<0.001) apoptosis, whencompared to control (**Figure 5A**). Similarly, in PANC1 cell line, BA (20 µM), TQ (25 µM) and GCB (5.25 µM), individually produced 9%, 8%, 7% rate of apoptosis, respectively. While in combination, CI _(GCB:BA)_, and CI _(GCB:TQ)_, the rate increased to 11% and 12% (p value<0.001), respectively when compared to control (**Figure 5B**). In combination treatment there was an increase in the rate of apoptosis in both the cell lines. BA produced more apoptotic effect individually and in combination with GCB than GCB and TQ alone or TQ in combination with GCB in MIA PaCa-2; while, reverse was observed in PANC-1 cells.

### PKM2 expression and Activity

**Figure 6:**
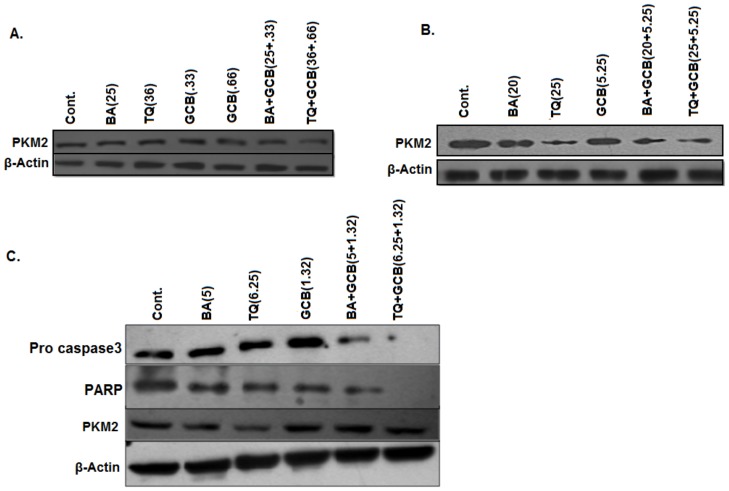
Protein expression analysis. *(A) MIA PaCa-2 and (B) PANC-1 synergetic doses at Fa 0.75 of Betulinic acid and Thymoquinone were used alone and in combination CI_(GCB:BA)_ and CI_(GCB:TQ)_ with Gemcitabine.* *From the blots it was observed that there was a decrease in expression of PKM2 when treated in combination doses. (C) PANC1 cell line doses at Fa 0.5 of betulinic acid, Thymoquinone and Gemcitabine were used alone and in combination, and from the blots we observed that there was a decrease in Pro-Caspase-3 followed by decrease in PARP and a slight decrease is observed in Thymoquinone treated in PKM2.*

**Figure 7:**
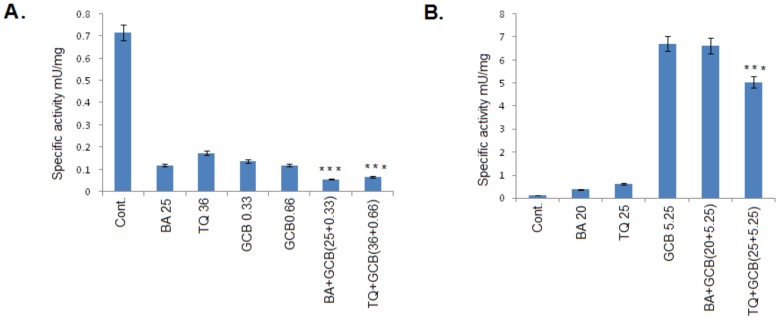
Specific activity of Pyruvate Kinase (PK)-M2 in both of human pancreatic tumor cell lines. *(A) MIA PaCa-2 cells treated with Betulinic acid, Thymoquinone and Gemcitabine alone and in combination, CI_(GCB:BA)_ and* *CI_(GCB:TQ)_ at 48 hrs. A decrease in the activity in treated cells followed with* *more* *decrease* *in combination(s), CI_(GCB:BA)_* *and CI_(GCB:TQ)_,* *as compared with the control was observed;* *(B)* In PANC-1 cell line vice versa was observed. Control versus monotherapy (BA, TQ, GCB) and Control versus combinations (CI_(GCB:BA)_ & CI_(GCB:TQ)_), (***, significant difference at p value<0.001, n = 3)

BA or TQ pre-treatment with a synergistic dose of Fa 0.75 along with GCB in combination (CI_GCB:BA_ and CI_GCB:TQ_) for 48 hrs in both pancreatic cancer cell lines, MIA PaCa-2 and PANC-1, showed a decrease inPKM2 protein level (**Figure 6A and B**). While, at Fa 0.5 i.e. cells pre-treated with BA (5 µM) and TQ (6.25 µM) in combination with GCB (1.3 µM) for 48 hrs, a decrease was observed in Pro-Caspase-3 expression and PARP; without any effect on PKM2 expression, except for a moderate decrease in the expression in PKM2 with TQ (**Figure 6C**). The activity of PKM2, however, showed an increase inuntreated MIA PaCa-2 cells and a decrease in the treated cells with BA (25 µM), TQ (36 µM) and GCB (0.33 µM and 0.66 µM); which enhanced in the combination (CI_GCB:BA_ and CI_GCB:TQ_) (**Figure 7A**). While, in case of PANC-1 cells, the results so obtained were reverse in un-treated cells which showed a decreased activity of PKM2 and an increase in the activity in the treated cells alone or in combination (**Figure 7B**).

## Discussion

Pancreatic tumors that are refractory to Gemcitabine therapy pose a challenge to oncologists. Therapies which are currently in use for treatment of pancreatic cancer have shown disappointing efficacy; and the acquisition of drug resistance during chemotherapy constitutes a major challenge. Thus, new strategies need to be put in place in order to develop novel chemo-preventive and/or chemo-therapeutic agents that would improve the clinical outcome of this disease. Growing evidence encourages the use of dietary molecules and supplementation within the diet to augment the response of standard cancer chemotherapeutic agents. Previously, flavonoids have been reported to inactivate frequently deregulated pathways, such as Akt and NF-κB, in pancreatic cancer, contributing to cell growth, metastasis and chemo resistance. Genistein, a dietary compound, has been shown to enhance the anti-tumor activity of erlotinib and gemcitabine in experimental systems of pancreatic cancer [40]. Earlier, it has also been demonstrated that Garcinol, a dietary molecule, synergizes with gemcitabine to inhibit cell proliferation and induces apoptosis in MIA PaCa-2 cells with significant modulation of key cancer regulators, including *PARP*, *VEGF*, *MMPs*, *ILs*, caspases, and *NF-κB* [*41*]. Curcumin, a dietary component, sensitizes pancreatic cancer to gemcitabine *in vitro* and *in vivo. In vitro*, curcumin was shown to inhibit the proliferation of various pancreatic cancer cell lines, potentiating apoptosis induced by gemcitabine, and inhibiting constitutive NF-κB activation in the cells. *In vivo*, tumors from nude mice injected with pancreatic cancer cells and treated with a combination of curcumin and gemcitabine showed a significant reduction in volume (*P* =  0.008 vs control; *P* =  0.036 vs gemcitabine alone) [42]. In our experiments, a combined interaction of betulinic acid (BA) or thymoquinone (TQ), the two neutraceuticals with gemcitabine (GCB), in GCB-sensitive-MIA PaCa-2 and GCB-resistant-PANC-1 - pancreatic cancer cell lines, showed synergism. The viability assay revealed a synergistic cytotoxic interaction between GCB and BA or GCB and TQ in both GCB-sensitive and -resistant cell lines, as determined by the DRI (Dose Reduction Index) of >1 obtained from calcusyn [43]. Mechanistically, GCB mediates its cytotoxic effects by inhibiting the repair synthesis step through its action as a ribonucleotide-reductase inhibitor, depleting the intracellular deoxy-nucleotide pools, and enhancing the potential of its own incorporation into newly synthesized DNA. Once incorporated into DNA, the analog causes termination of DNA synthesis and is resistant to removal by exo-nucleases, resulting in DNA strand breaks [44]. Betulinic acid (BA), a nutraceutical, has been shown to induce apoptosis through mitochondrial pathways, where during this process both the mitochondrial outer and inner membranes are permeabilized, resulting in release of soluble proteins, such as cytochrome c, Smac or AIF, from the mitochondrial interspace into the cytosol [45]. BA also induces deathin several different cancer cell lines through multiple pathways, which include p53-independent induction of p21/Waf1,up-regulation of death receptors, inhibition of specificity protein(Sp) transcription factors [46]. Similarly, Thymoquinone (TQ) induces apoptosis in tumor cells by several mechanisms, including NF-kB suppression, Akt activation and extracellular signal-regulated kinase signaling pathways, and also by inhibiting tumor angiogenesis [47]. On the basis of ED_50_, ED_75_ and ED_90_ of drug combinations, isobolograms were generated and the synergy evaluated, using CalcuSyn. Betulinic acid (BA) or Thymoquinone (TQ) in combination with gemcitabine (GCB), at multiple drug concentrations, showed that in GCB-sensitive-MIA PaCa-2 and GCB-resistant-PANC-1 cells, the combination index (CI)was under 0.88 at Fa of 0.75 (75% reduction of cell growth) in MIA PaCa-2 cells. Whereas, in case of PANC-1, the combination index (CI) was under 0.76 at Fa of 0.5. The biological basis for this synergy was clear in differential changes in apoptosis with both the dietary molecules. Even though mechanistically our combinations are “mutually non-exclusive” in nature but both the dietary molecules showed a significant difference in quantum of cytotoxicity induction in comparison to stand alone GCB. However, the rate of apoptosis induction by BA in combination with GCB was more efficient in both the cell lines, than TQ in combination with GCB. Also, decrease in the trans-membrane mitochondrial potential (Ψ) was observed when BA alone or in combination with GCB was provided to both the cell lines. No change, however, was observed on trans-membrane mitochondrial potential (Ψ) when cancer cell lines were treated with GCB alone. PANC-1, which is resistant to GCB than MIA PaCa-2, did not decrease the trans-membrane mitochondrial potential (Ψ) more efficiently in our experiment with BA in combination with GCB.

It was surmised that targeting the glycolytic regulation may be an effective strategy for a shift from predominantly mitochondrial metabolism toward glycolysis. A shift toward glycolysis appears to confer a number of survival advantages for tumor cells. These include resistance to hypoxia, which is prevalent in pancreatic tumors and an increased availability of glycolytic intermediates for use in the anabolic pathways that drive cell proliferation. Cancer cells utilize glucose at higher rates than normal tissue, and use most of glucose for glycolysis even under normoxic condition, which is known as the Warburg effect. Pyruvate kinase (PK) catalyzes the last step in the process of glycolysis and one of its isoforms (PKM2) has been reported to be associated with tumor progression, promoting the Warburg effect in cancer cells associated with increased glucose uptake and increased lactate production with decreased oxygen consumption. Compromised PKM2 activity has been observed to shift preferentially towards anabolic metabolism, resulting in macromolecular biosynthesis, instead of leading to oxidative metabolism for energy production, which promotes cancer cell proliferation and tumor growth. PKM2, as a tuner, is upstream of the decision point between oxidative and glycolytic metabolism and the decreased catalytic activity of PKM2 is believed to shunt glycolytic metabolites towards PPP in cancer cells [48]. Targeting cancer metabolism, therefore, is receiving considerable attention as a potentially attractive strategy for cancer therapy. Because of many important roles played by PKM2 as a protein kinase and a transcriptional co-activator, the most effective therapeutic strategy for targeting the protein may be to prevent its synthesis, either at the transcriptional or translational level and exploit the metabolic difference between cancer cells and normal cells for therapeutic benefit. Here in this study, the outcome of the drug (GCB) exposure in combination with the dietary agents, BA or TQ, were analyzed on PKM2 expression and activity. It was observed that PKM2 expression was independent of the PKM2 activity; and that of Pro-Caspase-3. PKM2 down-regulation in cancer cells resulted in a dose dependent manner. No decrease in the expression of PKM2 was observed at low concentrations of drug combinations. The IC_50_ value combination of dietary molecules with the low fixed dose of GCB, decreased the expression level of PKM2 significantly. The results showed that pre-treatment with the dietary molecule *i.e*. Betulinic acid (BA) or Thymoquinone (TQ) synergized with the low-dose of Gemcitabine (GCB), and induced cell death. Our results have demonstrated that TQ, BA and GCB potentiate cytotoxicity across a range of clinically achievable doses, an inference drawn from the experiments with human pancreatic cancer cell lines. The clinical implications of this synergistic interaction are paramount and include improvements in efficacy, treatment-associated toxicity, tolerability of therapeutic regimens, and quality of life. This data also corroborates with prior studies, suggesting a synergistic interaction between TQ, BA and GCB, as investigative therapeutic regimens in the treatment of pancreatic cancer. Our observations add an important value in the possible clinical treatment with proposed combinations, minimizing the dose of GCB, and thereby decrease the risk of toxicity. It would, however, be interesting to find out in future if the synergistic effect is mediated through the down regulation of the expression of PKM2. Nevertheless, based on the observations, it is apparent that the combinations of Betulinic acid and Gemcitabine, or Thymoquinone and Gemcitabine may offer a promising new approach in effective treatment of clinically heterogeneous human pancreatic cancers.

## Supporting Information

Figure S1 **Representation of Fa-CI plots.** *(A) MIA PaCa-2 (B) PANC-1 Fa-CI plots of Betulinic acid with Gemcitabine and Thymoquinone and Gemcitabine: Doses below the additive line, represents the synergistic effect. Whereas the doses above the additive line, represents the antagonistic effect.*
(TIF)

Table S1 **Dietary molecule (BA & TQ) and Drug (GCB) concentration along with ED values of the isobologram.**
(DOCX)

## References

[1] Tempero MA, Berlin J, Ducreux M, Haller D, Harper P, et al (2011) Pancreatic cancer treatment and research: an international expert panel discussion. Ann Oncol 22: 1500–1506. 21199884 10.1093/annonc/mdq545

[2] Kang SP, Saif MW (2008) Pharmacogenomics and pancreatic cancer treatment. Optimizing current therapy and individualizing future therapy. JOP 9: 251–266. 18469437

[3] el-Kamar FG, Grossbard ML, Kozuch PS (2003) Metastatic pancreatic cancer: emerging strategies in chemotherapy and palliative care. Oncologist 8: 18–34. 10.1634/theoncologist.8-1-18 12604729

[4] Rajendran P, Ho E, Williams DE, Dashwood RH (2011) Dietary phytochemicals, HDAC inhibition, and DNA damage/repair defects in cancer cells. Clin Epigenetics 3: 4. 22247744 10.1186/1868-7083-3-4 PMC3255482

[5] Fulda S (2009) Betulinic acid: a natural product with anticancer activity. Mol Nutr Food Res 53: 140–146. 19065582 10.1002/mnfr.200700491

[6] Fulda S, Kroemer G (2009) Targeting mitochondrial apoptosis by betulinic acid in human cancers. Drug Discov Today 14: 885–890. 19520182 10.1016/j.drudis.2009.05.015

[7] Zuco V, Supino R, Righetti SC, Cleris L, Marchesi E, et al (2002) Selective cytotoxicity of betulinic acid on tumor cell lines, but not on normal cells. Cancer Lett 175: 17–25. 11734332 10.1016/s0304-3835(01)00718-2

[8] Padhye S, Banerjee S, Ahmad A, Mohammad R, Sarkar FH (2008) From here to eternity - the secret of Pharaohs: Therapeutic potential of black cumin seeds and beyond. Cancer Ther 6: 495–510. 19018291 PMC2583426

[9] Norwood AA, Tan M, May M, Tucci M, Benghuzzi H (2006) Comparison of potential chemotherapeutic agents, 5-fluoruracil, green tea, and thymoquinone on colon cancer cells. Biomed Sci Instrum 42: 350–356. 16817633

[10] Gali-Muhtasib H, Kuester D, Mawrin C, Bajbouj K, Diestel A, et al (2008) Thymoquinone triggers inactivation of the stress response pathway sensor CHEK1 and contributes to apoptosis in colorectal cancer cells. Cancer Res 68: 5609–5618. 18632613 10.1158/0008-5472.CAN-08-0884

[11] Shoieb AM, Elgayyar M, Dudrick PS, Bell JL, Tithof PK (2003) In vitro inhibition of growth and induction of apoptosis in cancer cell lines by thymoquinone. Int J Oncol 22: 107–113. 12469192

[12] Wilson-Simpson F, Vance S, Benghuzzi H (2007) Physiological responses of ES-2 ovarian cell line following administration of epigallocatechin-3-gallate (EGCG), thymoquinone (TQ), and selenium (SE). Biomed Sci Instrum 43: 378–383. 17487111

[13] El-Mahdy MA, Zhu Q, Wang QE, Wani G, Wani AA (2005) Thymoquinone induces apoptosis through activation of caspase-8 and mitochondrial events in p53-null myeloblastic leukemia HL-60 cells. Int J Cancer 117: 409–417. 15906362 10.1002/ijc.21205

[14] Roepke M, Diestel A, Bajbouj K, Walluscheck D, Schonfeld P, et al (2007) Lack of p53 augments thymoquinone-induced apoptosis and caspase activation in human osteosarcoma cells. Cancer Biol Ther 6: 160–169. 17218778 10.4161/cbt.6.2.3575

[15] Rooney S, Ryan MF (2005) Effects of alpha-hederin and thymoquinone, constituents of Nigella sativa, on human cancer cell lines. Anticancer Res 25: 2199–2204. 16158964

[16] Wu ZH, Chen Z, Shen Y, Huang LL, Jiang P (2011) [Anti-metastasis effect of thymoquinone on human pancreatic cancer]. Yao Xue Xue Bao 46: 910–914. 22007514

[17] Banerjee S, Kaseb AO, Wang Z, Kong D, Mohammad M, et al (2009) Antitumor activity of gemcitabine and oxaliplatin is augmented by thymoquinone in pancreatic cancer. Cancer Res 69: 5575–5583. 19549912 10.1158/0008-5472.CAN-08-4235

[18] Warburg O, Wind F, Negelein E (1927) The Metabolism of Tumors in the Body. J Gen Physiol 8: 519–530. 19872213 10.1085/jgp.8.6.519 PMC2140820

[19] Warburg O (1956) On the origin of cancer cells. Science 123: 309–314. 13298683 10.1126/science.123.3191.309

[20] Curi R, Newsholme P, Newsholme EA (1988) Metabolism of pyruvate by isolated rat mesenteric lymphocytes, lymphocyte mitochondria and isolated mouse macrophages. Biochem J 250: 383–388. 3128282 10.1042/bj2500383 PMC1148867

[21] Pfeiffer T, Schuster S, Bonhoeffer S (2001) Cooperation and competition in the evolution of ATP-producing pathways. Science 292: 504–507. 11283355 10.1126/science.1058079

[22] Wong N, De Melo J, Tang D (2013) PKM2, a Central Point of Regulation in Cancer Metabolism. Int J Cell Biol 2013: 242513. 23476652 10.1155/2013/242513 PMC3586519

[23] Christofk HR, Vander Heiden MG, Harris MH, Ramanathan A, Gerszten RE, et al (2008) The M2 splice isoform of pyruvate kinase is important for cancer metabolism and tumour growth. Nature 452: 230–233. 18337823 10.1038/nature06734

[24] Brinck U, Eigenbrodt E, Oehmke M, Mazurek S, Fischer G (1994) L- and M2-pyruvate kinase expression in renal cell carcinomas and their metastases. Virchows Arch 424: 177–185. 8180780 10.1007/BF00193498

[25] Schneider J, Neu K, Grimm H, Velcovsky HG, Weisse G, et al (2002) Tumor M2-pyruvate kinase in lung cancer patients: immunohistochemical detection and disease monitoring. Anticancer Res 22: 311–318. 12017309

[26] Wechsel HW, Petri E, Bichler KH, Feil G (1999) Marker for renal cell carcinoma (RCC): the dimeric form of pyruvate kinase type M2 (Tu M2-PK). Anticancer Res 19: 2583–2590. 10470199

[27] Oremek GM, Teigelkamp S, Kramer W, Eigenbrodt E, Usadel KH (1999) The pyruvate kinase isoenzyme tumor M2 (Tu M2-PK) as a tumor marker for renal carcinoma. Anticancer Res 19: 2599–2601. 10470201

[28] Pottek T, Muller M, Blum T, Hartmann M (2000) Tu-M2-PK in the blood of testicular and cubital veins in men with testicular cancer. Anticancer Res 20: 5029–5033. 11326662

[29] Luftner D, Mesterharm J, Akrivakis C, Geppert R, Petrides PE, et al (2000) Tumor type M2 pyruvate kinase expression in advanced breast cancer. Anticancer Res 20: 5077–5082. 11326672

[30] Roigas J, Schulze G, Raytarowski S, Jung K, Schnorr D, et al (2001) Tumor M2 pyruvate kinase in plasma of patients with urological tumors. Tumour Biol 22: 282–285. 11553857 10.1159/000050628

[31] Mazurek S, Boschek CB, Hugo F, Eigenbrodt E (2005) Pyruvate kinase type M2 and its role in tumor growth and spreading. Semin Cancer Biol 15: 300–308. 15908230 10.1016/j.semcancer.2005.04.009

[32] Hardt PD, Toepler M, Ngoumou B, Rupp J, Kloer HU (2003) Measurement of fecal pyruvate kinase type M2 (tumor M2-PK) concentrations in patients with gastric cancer, colorectal cancer, colorectal adenomas and controls. Anticancer Res 23: 851–853. 12820312

[33] Bluemlein K, Gruning NM, Feichtinger RG, Lehrach H, Kofler B, et al (2011) No evidence for a shift in pyruvate kinase PKM1 to PKM2 expression during tumorigenesis. Oncotarget 2: 393–400. 21789790 10.18632/oncotarget.278 PMC3248187

[34] Chou TC, Talalay P (1984) Quantitative analysis of dose-effect relationships: the combined effects of multiple drugs or enzyme inhibitors. Adv Enzyme Regul 22: 27–55. 6382953 10.1016/0065-2571(84)90007-4

[35] Zhao L, Wientjes MG, Au JL (2004) Evaluation of combination chemotherapy: integration of nonlinear regression, curve shift, isobologram, and combination index analyses. Clin Cancer Res 10: 7994–8004. 15585635 10.1158/1078-0432.CCR-04-1087

[36] Kumar A, Malik F, Bhushan S, Sethi VK, Shahi AK, et al (2008) An essential oil and its major constituent isointermedeol induce apoptosis by increased expression of mitochondrial cytochrome c and apical death receptors in human leukaemia HL-60 cells. Chem Biol Interact 171: 332–347. 18070620 10.1016/j.cbi.2007.10.003

[37] Lemasters JJ, Nieminen AL (1997) Mitochondrial oxygen radical formation during reductive and oxidative stress to intact hepatocytes. Biosci Rep 17: 281–291. 9337483 10.1023/a:1027332611839

[38] Bhushan S, Kumar A, Malik F, Andotra SS, Sethi VK, et al (2007) A triterpenediol from Boswellia serrata induces apoptosis through both the intrinsic and extrinsic apoptotic pathways in human leukemia HL-60 cells. Apoptosis 12: 1911–1926. 17636381 10.1007/s10495-007-0105-5

[39] Iqbal MA, Siddiqui FA, Gupta V, Chattopadhyay S, Gopinath P, et al (2013) Insulin enhances metabolic capacities of cancer cells by dual regulation of glycolytic enzyme pyruvate kinase M2. Mol Cancer 12: 72. 23837608 10.1186/1476-4598-12-72 PMC3710280

[40] Banerjee S, Zhang Y, Ali S, Bhuiyan M, Wang Z, et al (2005) Molecular evidence for increased antitumor activity of gemcitabine by genistein in vitro and in vivo using an orthotopic model of pancreatic cancer. Cancer Res 65: 9064–9072. 16204081 10.1158/0008-5472.CAN-05-1330

[41] Parasramka MA, Ali S, Banerjee S, Deryavoush T, Sarkar FH, et al (2013) Garcinol sensitizes human pancreatic adenocarcinoma cells to gemcitabine in association with microRNA signatures. Mol Nutr Food Res 57: 235–248. 23293055 10.1002/mnfr.201200297

[42] Kunnumakkara AB, Guha S, Krishnan S, Diagaradjane P, Gelovani J, et al (2007) Curcumin potentiates antitumor activity of gemcitabine in an orthotopic model of pancreatic cancer through suppression of proliferation, angiogenesis, and inhibition of nuclear factor-kappaB-regulated gene products. Cancer Res 67: 3853–3861. 17440100 10.1158/0008-5472.CAN-06-4257

[43] Chou TC (2010) Drug combination studies and their synergy quantification using the Chou-Talalay method. Cancer Res 70: 440–446. 20068163 10.1158/0008-5472.CAN-09-1947

[44] Plunkett W, Huang P, Gandhi V (1995) Preclinical characteristics of gemcitabine. Anticancer Drugs 6 Suppl 6 7–13. 10.1097/00001813-199512006-00002 8718419

[45] Fulda S (2008) Betulinic Acid for cancer treatment and prevention. Int J Mol Sci 9: 1096–1107. 19325847 10.3390/ijms9061096 PMC2658785

[46] Rabi T, Shukla S, Gupta S (2008) Betulinic acid suppresses constitutive and TNFalpha-induced NF-kappaB activation and induces apoptosis in human prostate carcinoma PC-3 cells. Mol Carcinog 47: 964–973. 18444250 10.1002/mc.20447 PMC2864721

[47] Kacem R, Meraihi Z (2009) The effect of essential oil extracted from Nigella sativa (L.) seeds on human neutrophil functions. Nat Prod Res 23: 1168–1175. 19731134 10.1080/14786410802228611

[48] Luo W, Semenza GL (2012) Emerging roles of PKM2 in cell metabolism and cancer progression. Trends Endocrinol Metab 23: 560–566. 22824010 10.1016/j.tem.2012.06.010 PMC3466350

