# Supplementary figures and images for: Synergistic Combination of Gemcitabine and Dietary Molecule Induces Apoptosis in Pancreatic Cancer Cells and Down Regulates PKM2 Expression
Source: PLoS One. 2014 Sep 8;9(9):e107154. doi: 10.1371/journal.pone.0107154 (PMC4157832; doi:10.1371/journal.pone.0107154)

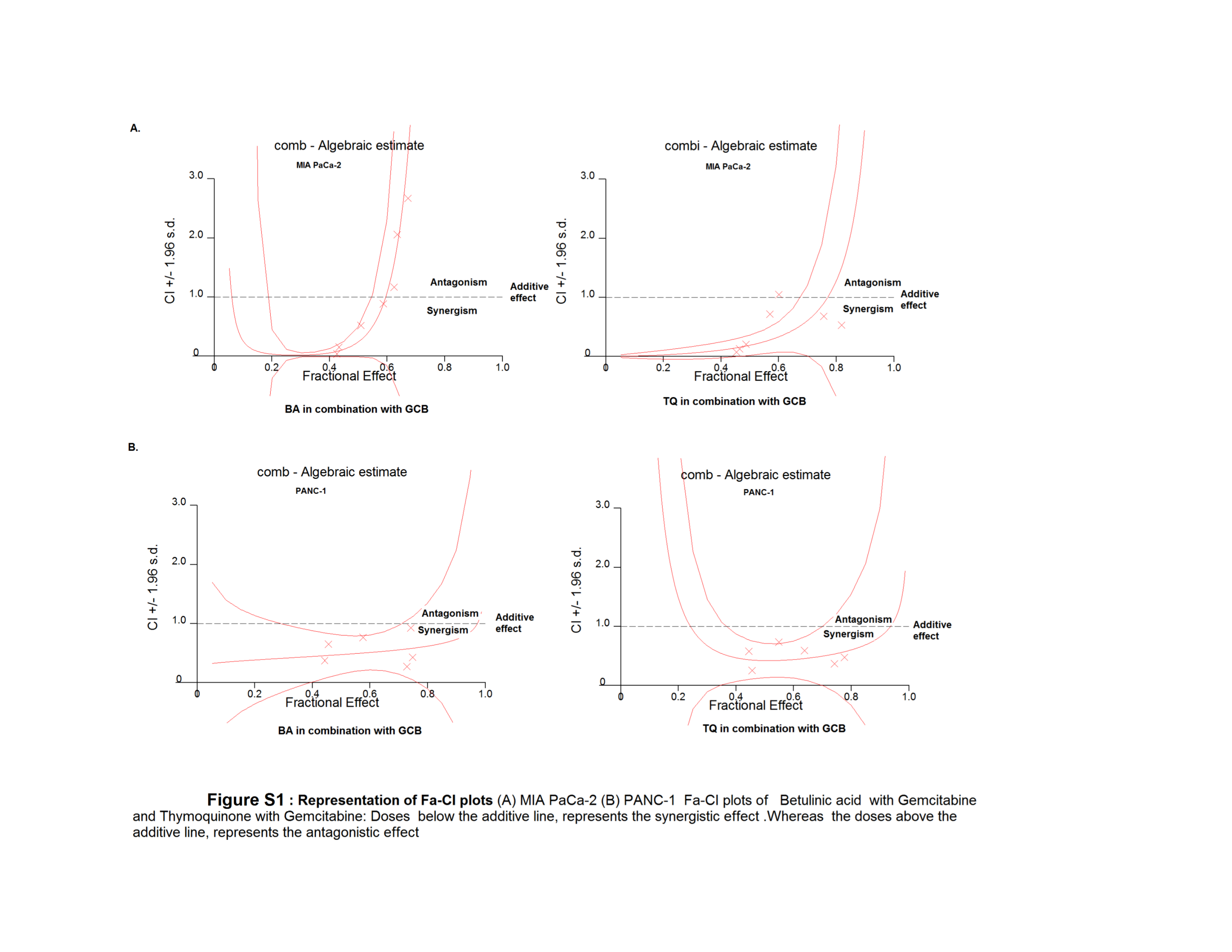

Supplement: Figure S1 — Representation of Fa-CI plots. (A) MIA PaCa-2 (B) PANC-1 Fa-CI plots of Betulinic acid with Gemcitabine and Thymoquinone and Gemcitabine: Doses below the additive line, represents the synergistic effect. Whereas the doses above the additive line, represents the antagonistic effect. (TIF) [file pone.0107154.s001.tif]
