# Supplementary material for: Synergistic Combination of Gemcitabine and Dietary Molecule Induces Apoptosis in Pancreatic Cancer Cells and Down Regulates PKM2 Expression
Source: PLoS One. 2014 Sep 8;9(9):e107154. doi: 10.1371/journal.pone.0107154 (PMC4157832; doi:10.1371/journal.pone.0107154)
Supplement: Table S1 — Dietary molecule (BA & TQ) and Drug (GCB) concentration along with ED values of the isobologram. (DOCX) [file pone.0107154.s002.docx]

**Supplementary Table 1: Dietary molecule (BA & TQ) and Drug (GCB) concentration along with ED values of the isobologram:**

| **Cell line** | **Dietary(μM) : Drug (nM)** | **ED values** |
| --- | --- | --- |
| **MIA PaCa-2** | ( BA:GCB)(10:30) | ED50 |
|  | ( TQ: GCB)(38:40) | ED90 |
| **PANC-1** | ( BA: GCB)(41:38) | ED90 |
|  | ( TQ: GCB)(48:37) | ED90 |
